# Virtual sample based techniques using deep features for SSPP face recognition in unconstrained environment

**DOI:** 10.1371/journal.pone.0322638

**Published:** 2025-05-22

**Authors:** Muhammad Tariq Siddique, Ibrahim Venkat, Humera Farooq, Sharul Tajuddin, S. H. Shah Newaz

**Affiliations:** 1 Department of Computer Sciences, Bahria University, Karachi, Pakistan; 2 School of Computing and Informatics, Universiti Teknologi Brunei, Jalan Tungku Link Gadong, Brunei-Muara, Brunei Darussalam; Universita Campus Bio-Medico di Roma, ITALY

## Abstract

As challenging as it is to use face recognition with a Single Sample Per Person, it becomes even more difficult when face recognition based on a single sample is performed in an unconstrained environment. The unconstrained environment is normally considered irregular in facial expressions, pose, occlusion, and illumination. This degree of difficulty increases as a result of the single sample and in the presence of occlusion. Extensive research has been done on face recognition under pose and expression changes. Comparatively, less research has been reported on the occlusion problem that occurs in facial images. Occlusion may alter the appearance of facial images and cause deterioration in recognition. A robust method is required to handle the occlusion in the face image to improve the recognition performance. This study aimed to implement an effective augmentation technique that improves the performance of the Single Sample Per Person face recognition system in unconstrained environments. Virtual samples were created to expand the sample size to address the problem of a single sample. A local region-based technique was proposed to deal with occlusion by creating virtual samples. A deep neural network-based model, FaceNet, was used to extract the features and a support vector machine was used for classification. The performance of the proposed approach was evaluated, demonstrating its superiority in handling occlusion compared to that of its state-of-the-art counterparts. The proposed method achieved significant accuracy improvements, specifically 94.83% for the occlusion with sunglasses and 98% for the occlusion with scarves in the AR dataset.

## 1 Introduction

Face recognition systems attempt to verify a subject from a video or image using facial information [1]. These systems possess the characteristics that make them unique from other biometric systems, and these qualities are contactless, nonintrusive, convenient, and scalable. These traits are used to identify and verify individuals. Face recognition is considered a prominent approach to the identification and surveillance of biometrics field users [2].

The Single Sample Per Person (SSPP) is a complex problem of single reference samples from facial databases. The goal is to recognize and identify a person considering interrelated information along with unpredictable poses, geometric and photometric changes, facial expression, age, illumination, low resolution, dimensionality, occlusion with accessories, makeup, and hairstyle from one sample [3]. Variations caused by the unconstrained environment in facial appearance have a greater effect than personal identity [4]. In particular, face images of different subjects look similar compared to the same subject’s face image taken under varied conditions in an unconstrained environment [5]. The researchers found it a challenging problem with most current algorithms due to less training data and low dimensionality [6].

Since the face recognition problem is considered noninvasive, it could go through the occlusion problem. The most common occlusion for subjects uses different accessories such as sunglasses, scarves, and masks to partially hide their facial regions. The objects in between the camera and the face also create occlusion, and this causes the loss of certain parts of the information. Local region-based methods are used to solve the occlusion problem in SSPP face recognition. The works of [7,8] show the effectiveness of these methods in occlusion. These methods also proved that face recognition performance is more affected by the upper region compared to the lower region occlusion.

In addition, to address the above-mentioned issues, a large training set is required, especially for Deep Learning (DL) approaches. In the past decade, DL approaches have been extensively applied to enhance Face Recognition (FR) models[9] and several efforts have been made to handle the Single Sample Per Person Face Recognition (SSPP FR) problem under controlled conditions [10]. However, the existing literature shows that there is still scope to investigate and deploy machine learning approaches to handle uncertainty elements encountered in unconstrained environments. Recently, a survey has been conducted for the utilization of DL approaches for SSPP [9]. Google [11] and Facebook [12] have played an important role in understanding the importance of the availability of large training data sets to utilize DL approaches for FR in an uncontrolled environment effectively. These challenges are mainly due to the non-rigid structure of the face, capturing conditions, and modeling of face recognition problems. In addition, when the environment has uncontrollable conditions, the system performance significantly decreases and severely limits the success of the identification.

Local region-based augmentation works independently on different features of the face. The local region-based augmentation technique has been widely used in the past and several studies have reported successfully treating facial expression, pose, and occlusion [6,13]. Efforts are still underway to overcome the issue of occlusion, that is, sunglasses and scarf occlusion in an unconstrained environment. Some examples are [14] and [7] using local region information to solve the occlusion problem in an unconfined environment. Facial images were divided into non-overlapped patches and statistical descriptors and histogram methods were used for feature extraction [14]. In [7], after dividing images into nonoverlapping patches, the eigenvalue was used to extract features in the form of matrices. In another method, facial information was divided into bilateral symmetry. The projection matrices method was used to extract the features [15].

The proposed study aims to identify and provide a solution to the problem of a small training set that is putting in place an effective enhancement technique. Knowledge of the right augmentation technique to enhance the training set is assumed to result in improved recognition performance. This information led us to propose a local region-based augmentation technique to solve the occlusion problem. The proposed approach requires a small number of augmented training samples and hence has the advantage of minimizing the training overhead of the classifier models.

## 2 Related work

Classical augmentation techniques are used to learn facial features as a global region for the intra-class variation and to increase the sample size for training by generating virtual samples [15–18]. On the other hand, the local region-based augmentation techniques are used to learn inter-class variations to increase the sample size virtually [6,7,14,15,19]. Similarly, deep learning and image processing techniques are also used to improve image quality and expand the training set for SSPP [20,21]. The generative techniques include Generative Adversarial Networks to produce synthetic images. For this purpose, various transformations are applied to the face images. Different existing studies based on the above-mentioned techniques are presented in Table 1 and discussed in the following subsections:

**Table 1:** Existing studies using different techniques to solve SSPP problem where P = Pose, E = Expression, I = Illumination, LR = Low resolution, O = Occlusion.

Approaches	Year and Studies	Augmentation Techniques	Feature Extraction	Classifier	Variations
Classical Augmentation Techniques	(3D Model) [24], 2024	3D Model	Deep Learning		P
	(WPD-HOG-P) [17], 2022	Bit plane, Sliding window, and Mirror transform	Image Pyramid,eak Wavelet Packeteak Decomposition,eak HOG	SVM	P, I , E
	(3D Model) [25],eak 2020	3D Model	Deep Learning		P, E ,I
	(SSLD) [6], 2019	Geometric Transformation (Translate and Scale)	VGG-16	k-LiMAPS algorithm	P, E, O, LR
	(DSFS) [47], 2018	3D face reconstruction	Dictionary Learning	Sparse based classification	P, I
	(PAML) [48], 2018	3D Model	Linear Regression	Metric Learning	P, I
	(CBM) [18], 2018	Virtual Images	Discriminative common vectors	kNN	I
Generative Adversarial Network	(SharedGAN)eak [29], 2022	Generic Dataset	CNN		P, E, LR, O, I
	(VD-GAN) [30], 2021	Generator and Discriminator learning	Autoencoder	kNN	P, I, E,O
	(IL-GAN) [28], 2019	Autoencoder	Autoencoder	CNN	I
	One-shot face Recognitioneak [31], 2018	Generic DataSet	CNN		P, E,I
Local Based Methods	(DNNC) [7], 2021	Eigen Values	NNC		P, I, O, E
	(SOM-BC) [38], 2021	Bag of Features	Self Organizing Maps		I, E,O
	(MB-C-BSIF) [14], 2021	BSIF	kNN		P, I, E, O
	(MMHD) [45], 2021	Edge Pixels	Hausdorff Distance		P, I, E, O
	(ADDL) [39], 2020	Auxiliary dictionary	Minimal regularized reconstruction Error		P, I, O
	(MFSA) [15], 2019	Projection Matric	kNN		P, I, E, O

### 2.1 Classical augmentation techniques

The augmentation techniques are used to artificially expand or increase the data volume for the training model [22]. It is widely used in the SSPP FR problem to enhance the available sample into multiple sets. The augmentation techniques are broadly divided into classical techniques and generative techniques [23]. The classical techniques include geometric transformation, which is divided into variations like rotation, image translation, noise injection, flipping and cropping, and photometric transformation which deals with light and shadow problems. Another study reported that face recognition is performed using infrared images for an unconstrained environment [24]. A detailed survey by X. Wang discussed the challenges and future directions for augmentation techniques for face datasets [3].

Recently, 3D Modeling [25] has been proposed that used augmentation techniques. In another study, virtual samples [17] were created using the Non-Negative Matrix Factorization (NMF) method. They used extension methods such as sliding windows, mirror transform, and bit plane. A k-LiMAPS algorithm was proposed that was based on optimal direction and iterative *l*_0_-norm minimization to substitute the Sparse Dictionary Learning (SSDL) technique. They worked on Geometric Transformation (Translate and Scale) [6]. Addressing the same problem, in a study different photometric and geometric transformations (rotating, flipping, cropping, edge enhancement, color jittering, and addition of principle components to the images) were used to increase the classification performance of CNN [26].

However, the classical techniques are unable to handle the diversity of face images in an unconstrained environment. Further, the training samples generated by the classical augmentation techniques could be easily correlated with the gallery images. This limitation degrades the classification as they could not be considered autonomous samples. In addition, the greater number of generated virtual samples maximizes training cost [6].

### 2.2 Generative methods

Generative methods are used to generate synthetic images to enlarge the training dataset. According to a survey paper [27], the generative methods enrich the gallery set from the available single reference face image by generating new synthetic images. The Generative Adversarial Network (GAN) has gained popularity due to its image restoration ability, especially in SSPP problems [28,29]. The method (IL-GAN) introduced by [28] based on GAN and a variational autoencoder. The singular value decomposition was used to create a decision-maker to distinguish between illumination levels. One method employed by [29] was based on creating virtual samples using GAN (SharedGAN). They trained the GAN on a generic dataset and used these variations in the gallery set. They have used a Convolutional Neural Network (CNN) for feature extraction and classification. Further, the different variations of the contaminated dataset were addressed by proposing a Variation Disentangling Generative Adversarial Network [30]. The proposed framework was based on the generator and discriminator that work in an adversarial way. In [31] by applying the GAN model, addressed the data imbalance problem in face recognition and proposed a large-scale system to deal with the pose, expression, and illumination. In the study [32], the authors have created samples using a generic dataset and error coding technique. They extracted errors from the generic set and perform multilevel error coding. An identity-attribute disentanglement framework has been introduced by [33] that separates identity-related and identity-irrelevant features to improve recognition accuracy. An adversarial feature augmentation mechanism was employed to generate diverse identity-preserving samples, enhancing model generalization. Recently, a conditional GAN proposed to generate synthetic face images from a single real sample. The method modifies attributes such as expression, age, gender, pose, and lighting to create diverse images while preserving the original identity [34].

### 2.3 Local region based techniques

The local region-based methods are used to solve the occlusion problem in SSPP face recognition. Different surveys conducted on SSPP FR highlight the working and effectiveness of local region-based methods [1,35]. To apply local region-based methods, several studies were reported in the past that proposed different techniques to learn facial information of the face and enhance small training data to generate robust results. To address the complex variation in an unconstrained environment the authors proposed methods like dictionary learning [36–40], Fuzzy Multi-Manifold Classifier [8], patch-based methods [41–43], and sparse representation classification [44] which are based on the local region or intra-class variation. These methods also proved that face recognition performance is more affected by the upper region than by the lower region occlusion. Another idea is to apply local region-based methods that are considered effective and low computational cost solutions for face recognition.

By using local region-based methods, most of the existing techniques work on the different variations of different existing datasets. A combination of different methods such as Auxiliary dictionary [39], Eigen Values [7], Projection Matric [15], Multi-Block Color-Binarized Statistical Image Features (MB-C-BSIF) [14], and Edge Pixels [45] are the recent studies that work on a pose, expression, occlusion, and illumination. Facial images were divided into non-overlapped patches and statistical descriptors and histogram methods were used for feature extraction [14]. In their extended work, they used VGG 16 for Feature Extraction and improved their results [13]. In [7], the Eigenvalue is used to extract features in the form of matrices after dividing images into non-overlapping patches. In another method, the facial information was divided into bilateral symmetry. The projection matrices method was used to extract the features [15]. A Self-Organizing Maps (SOM)-based technique [38] achieved good accuracy as compared to the existing studies, however, the usage of Scale-Invariant Feature Transform (SIFT) descriptors for extracting the local features may increase the computational time and cost. A dual-feature classification approach (DF-SRC) has been proposed that improves face recognition under occlusion. It combines global features from Discrete Wavelet Transform (DWT) and local features from Local Binary Patterns (LBP) for better representation. A sparse representation-based classification (SRC) method then reconstructs and classifies the face, making recognition more robust [46].

However, a closer look at the existing literature on SSPP problems based on local region-based methods reveals several gaps and shortcomings in the unconstrained environment. The local region-based methods provide solutions for inter-variance and intra-variance class problems in an unconstrained environment. Local region-based methods can lessen the effect of moderate facial variations. The image variations have a deep impact on local feature extraction and discriminative learning from partitioned patches. Misalignment or pose variations in probe samples cause a mismatch between the gallery and probe samples. The limitation of these methods lies in the complexity of variations. Patch-based methods are considered traditional methods that work on the local region of the faces. The main issue with patch-based methods is the presence of face misalignment that causes degradation of the recognition rate. The proposed study will use the local regions to solve the occlusion problem using the augmentation technique. The aim is to use this technique to increase the recognition performance of the SSPP FR using the Support Vector Machine (SVM) classification method.

## 3 Proposed augmentation techniques for SSPP in unconstrained environment

The presented approach aims to apply augmentation techniques to generate virtual samples to solve the problem of a small training set for the SSPP. We have applied the local region-based technique. The key points of facial images have been extracted from the eye and mouth region. While dealing with the occlusion in the facial images, we have proposed two-fold solution. To deal with the face occlusion problem the facial key points are detected first and ROI is extracted. After this, an overlay patch has been used to create artificial augmentation that is used as a virtual sample.

**Fig 1:**
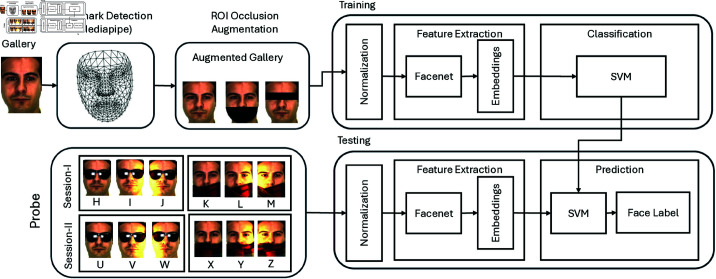
Proposed approach for SSPP FR problem using augmentation techniques.

The facial features are then extracted to train the classifier, and the test samples are classified. The pre-trained FaceNet model is used for normalization and face embedding. For classification purposes, we have used the SVM. The virtual sample will be used as a training set (gallery images) whereas testing will be done by the probe images. The complete methodology of the proposed study is shown in Fig 1.

### 3.1 Local area region-based technique

The local region-based methods are used to solve the occlusion and illumination and pose estimation problems in SSPP FR in an unconstrained environment. Local region-based augmentation works independently on different features of the face. We extracted the facial features and regions of interest (ROI) and used this information to recognize the face. For this purpose, the first step is the landmark detection method. This method is used to localize the important points and select ROI at the eye and mouth region of the gallery images. In the next step, an overlay patch was used in the ROI region of the gallery images. In the last step, artificial occlusion has been introduced to increase the sample size and thus create an augmented gallery. These steps are shown in Algorithm 1:

**Algorithm 1.** Local region-based augmentation.

**Input:** *I* (Gallery Image)

**Output:** *I*_*aug*_ (Occluded Augmented Images)

- Perform face detection to identify the face region
  - *R*_*f*_ = Facedetection(I)
- Detect facial landmark points and extract the region of interest (ROI)
  - *L*,*R*_*ROI*_ = Mediapipe(*R*_*f*_)
- Apply an overlay patch *P* to the *ROI*
  - *R*_*occluded*_ = *R*_*ROI*_ ⊕ *P*
- Create occluded images by replacing *R*_*ROI*_ in *I* with *R*_*occluded*_
  - *I*_*occ*_ = *I* - *R*_*ROI*_ + *I*_*occluded*_
  - Generate multiple occluded images
  - *I*_*aug*_ = {*I*_*occ*1_,*I*_*occ*2_,,*I*_*occm*_}
- Train the model using *I*_*aug*_
  - Extract features
  - *F* = *F*_*e*_(*I*_*a*_*ug*)
  - Classify features
  - *y* = C(*F*)

### 3.2 Facial keypoint detection

For local region-based augmentation, the landmark points detection method has been used. We have utilized the MediaPipe framework for this purpose [49,50]. The MediaPipe is developed by Google as an open-source framework and is available as a library for customization. This framework aims to provide a solution for different problems that could require an extensive computational cost. It could help design machine learning models for different objects using sensory devices. It helps to detect and track the key points of the human body known as landmark points and creates a face mesh. The face mesh is based on transfer learning and is designed to recognize the human face in three dimensions.

**Fig 2:**
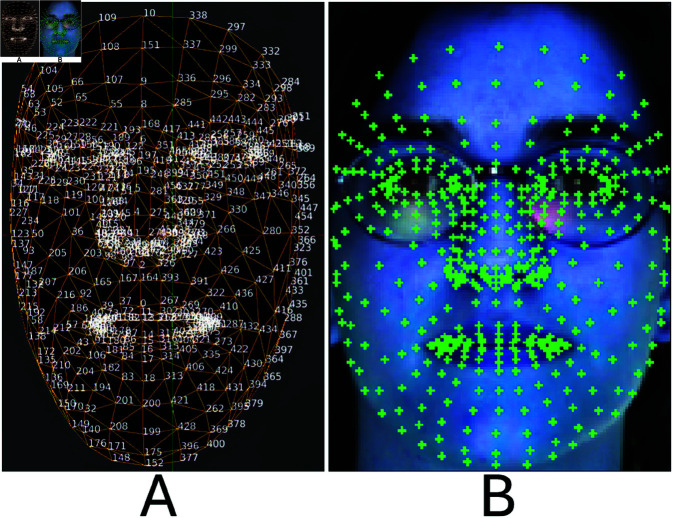
Landmark points of MediaPipe Face Mesh (A) Landmarks points (B) Landmarks points at AR dataset image.

The landmark points shown in Fig 2A are the provided information by FaceMesh and Fig 2B shows landmark points in the AR dataset image. We have used 04 landmark points (P1 to P4) out of 468 landmark points for the eyes region. For the mouth region, there are 19 landmark points (P1 to P19). These landmark points are obtained with the reference code provided in Fig 2A. The points used in this study with the referenced MediaPipe landmark points for the eye region are represented in Table 2. Whereas, Table 3 lists down the landmark points for the mouth region.

**Table 2:** Landmark points for the Eye Region.

Coordinates Points	MediaPipe Landmark ID	Description
p1	127	Extreme Left
p2	389	Extreme Right
p3	105	Eye Brows Area
p4	5	Below Eye Area

**Table 3:** Landmark points for the Mouth Region.

Coordinates Points	MediaPipe Landmark ID	Coordinates Points	MediaPipe Landmark ID
p1	93	p11	377
p2	132	p12	400
p3	58	p13	378
p4	172	p14	379
p5	136	p15	365
p6	150	p16	397
p7	149	p17	288
p8	176	p18	361
p9	148	P19	323
p10	152	-	-

**Fig 3:**
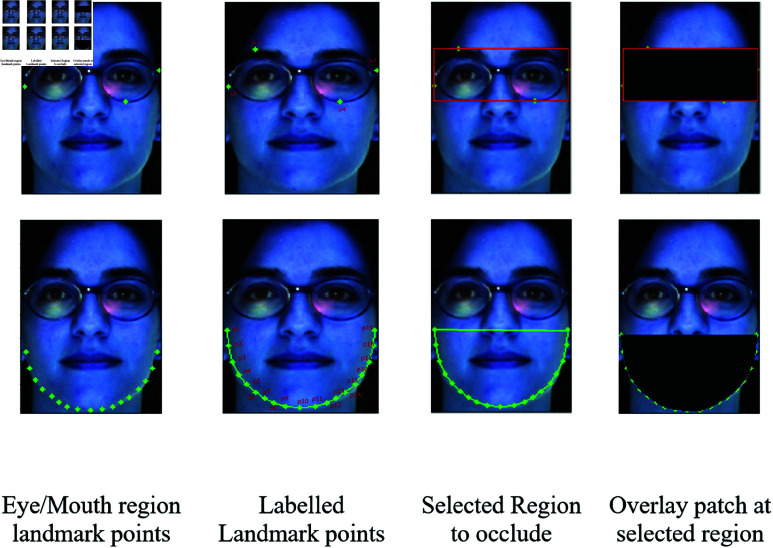
An example of AR dataset image for retrieving landmark points and location.

The selected landmark points are shown in the first row of Fig 3. These 19 landmark points and their location in the mouth region are shown in the bottom row of Fig 3. Other than these 19 landmark points, these labeled key points, a selected region, and an overlay patch at the selected region are also described. Given the input image, different face regions are detected according to the extreme points.

For a gallery image, the landmark set can be described as in Eq 1

$$\alpha_{i}=x_{i},y_{i},z_{i}$$ (1)

Where α represents the position of each landmark, i represents the landmark points from 1–468, and x, y, and z are the coordinates. Since in the proposed study, the face images are 2D that is why we utilized x and y landmark points and excluded the z coordinate.

For example, for the overall eye region the extreme left and right, where p1 represents the extreme left and p2 represents the extreme right. The eyebrows are represented by p3 and the below eye is represented by p4. Hence these four coordinates provide us with the region of interest for the eye region. Similarly, the calculated region of interest for the mouth is along the left and right cheeks starting from landmark point 93 until 323 with a total of 19 landmark points.

### 3.3 Artificial occlusion augmentation

**Fig 4:**
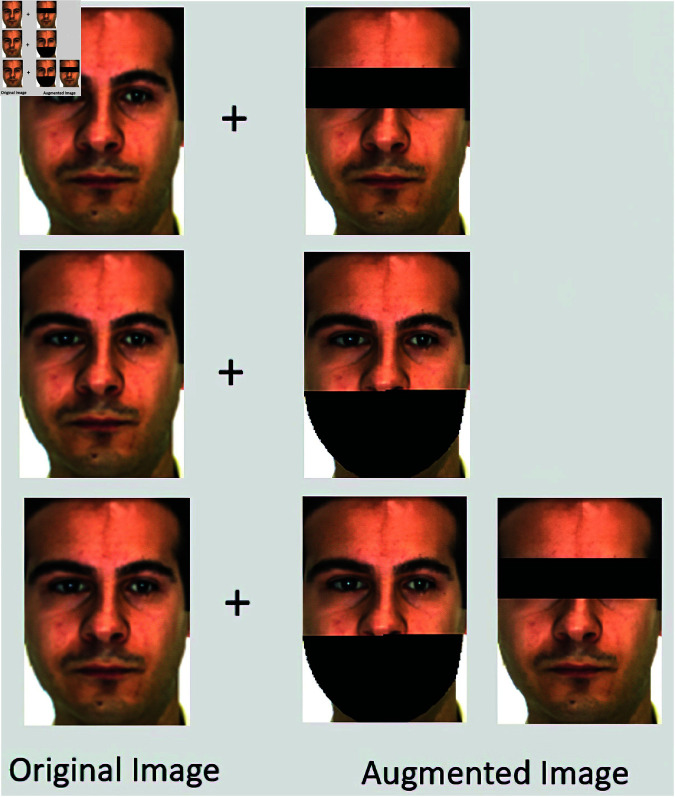
AR training dataset with original and augmented images.

Artificial occlusion augmentation is a major step in the training phase after the landmarks’ points detection. The region of interest is extracted to occlude the specific facial part. For the proposed study, both eyes and mouth are occluded to embody the sunglasses and mask or scarf occlusion of faces. For generating artificial occlusion, the gallery images of each subject have been extended. To apply the augmentation technique for creating artificial occlusion an overlay patch in a rectangular shape has been used. This overlay patch is used to occlude the eyes and mouth region. For the overlay patch, the α value has been used that represents two extreme points i.e. (0,1), where 0 value represents the dark region and 1 value represents the transparent region. For the eye region, the major occlusion is because of wearing sunglasses. Subject to the sunglasses, for the overlay patch the α having 0 value represents the sunglasses whereas 1 for the transparent region. Moreover, for the mouth region, the major reason for occlusion is wearing of mask and scarf. To deal with it the overlay patch has been introduced over the mouth region in which the α value 0 represents the patch like a mask or scarf and 1 represents the transparent region. After this extension, the gallery images are used to train the model as shown in Fig 4. Furthermore, the probe images that have real-time occlusion are used as a testing set.

### 3.4 Feature extraction, face embedding, and classification

After pre-processing images, the next step is to do the feature extractions of gallery and probe images. To extract the features, we have used a Deep Neural Network (DNN). There are two steps in this process. The first one is normalization and the second one is face embedding. Prior to the extraction of face embedding Z-Score normalization is utilized to standardize the image where the normalized image is obtained according to the following Eq 2.

$$PixelValue_{new}=\frac{PixelValue-\mu }{\sigma }$$ (2)

Where μ and σ are the mean and standard deviation of image pixel values.

For the face embedding process, we used a pre-trained FaceNet model [11]. FaceNet is an integrated system for face recognition, and it is also known as the Siamese network. It is a one-shot model, having the same size as the input images. The image input size is 160x160x3 which transforms into a 128-dimensional face embedding vector. The convolutional layers are used to learn facial features as mapping. Later on, the triplet loss function is used to calculate the face similarity using Euclidean distance. At last, the embedded feature vectors are extracted that are used for face recognition and verification [51].

The feature classification phase leads to face image verification and recognition. Verification compares a test image to other face images to approve the authentication of the requested face while recognition compares a test image with other images to come up with the identity of the face with several possibilities. In both scenarios, the known face images registered in the system are called gallery images. The face images either registered or unregistered used for testing are called probe images. Since our dataset is small in size so in the presented study, we have used SVM for the classification. The extracted features from the training face images in the dataset are utilized to train an SVM classifier. Once trained, the SVM classifier predicts the features of the test face images. SVM is chosen due to its superior interpretability and computational efficiency compared to other classifiers. Additionally, SVM demonstrates strong generalization capabilities, particularly in handling challenges such as the Single Sample Problem, where only limited training data is available for certain classes. Furthermore, SVM’s ability to find an optimal hyperplane for classification makes it well-suited for high-dimensional feature spaces, as often encountered in face recognition tasks. By employing kernel functions, SVM effectively maps non-linearly separable data into higher dimensions, improving classification accuracy. This flexibility, combined with its robustness against overfitting, enhances the reliability of SVM in real-world face recognition applications.

## 4 Experimental results

The proposed technique is used to extract the key points from the eye and mouth region as explained in detail in the methodology section.

### 4.1 Experimental setup

For local region-based augmentation techniques, the AR database [52] has been used because of the reason of the availability of occlusion. While selecting the dataset, the following criteria were taken:

a The existing datasets should have face images captured on realistic cases with varied challenges in unconstrained conditions.
b The actual image size was sampled down to the same size (160x160x3) for the experimental setup

**Fig 5:**
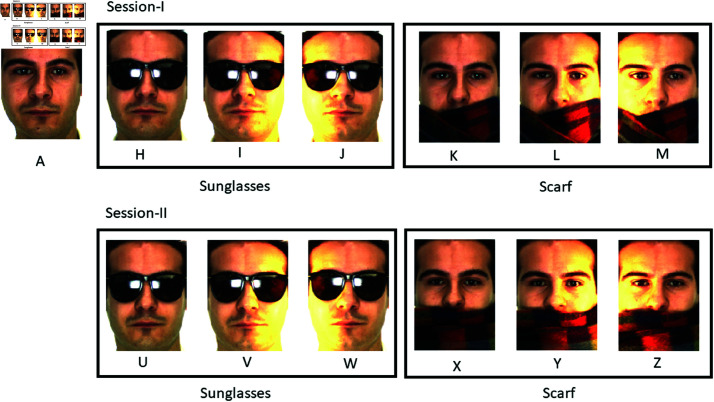
Representation of used sets from AR dataset.

The AR dataset has the combination of sunglasses and a scarf over the face occluded images. In the AR dataset, the original image resolution is 768*X*576 pixels. We down-sampled the original images into 160*X*160 pixels. For the experiments, we selected 100 subjects with 26 images. Among these 50 subjects are male and 50 subjects are female. The images of Set A in Session I having a neutral facial expression are selected as the gallery image as shown in Fig 5. For testing purposes, we selected test sets H- M and U-Z from Session I and II as follows:

1. S-I Occlusion with Sunglasses (H, I, and J)
2. S-I Occlusion with Scarf (K, L, and M)
3. S-II Occlusion with Sunglasses (U, V, and W)
4. S-II Occlusion with Scarf (X, Y, and Z)

To evaluate the model, the classification results have been validated through the accuracy. We have also demonstrated misclassification errors by taking sample results from each dataset. The results evaluation is performed based on the test accuracy. These experiments were performed on Intel Core i7-8750H with a 2.2 GHz processor and 16GB of memory. We used the Keras framework to load the FaceNet model for the experiments.

### 4.2 Occlusion with sunglasses

**Fig 6:**
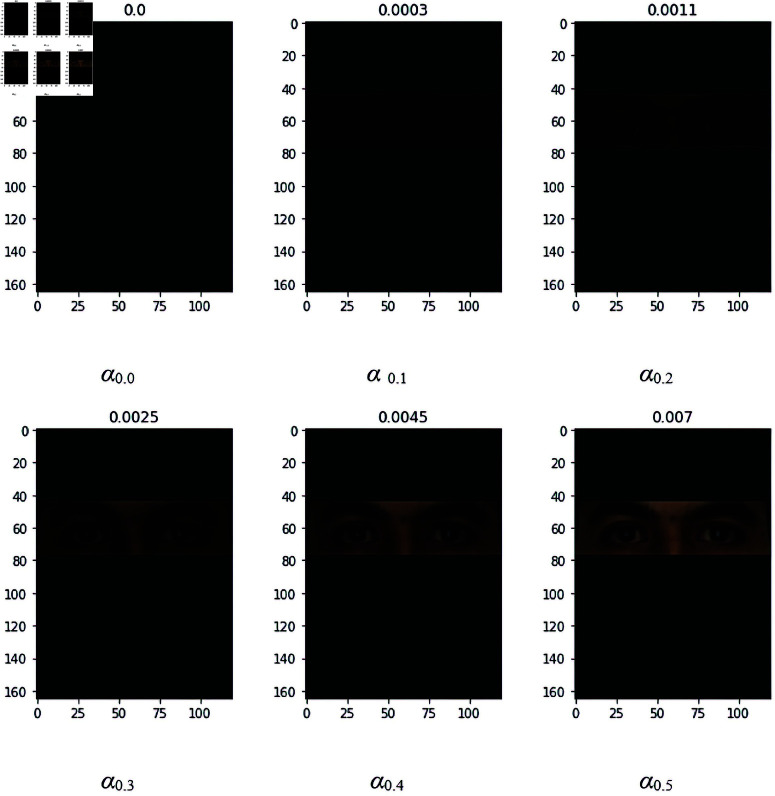
Occluded Image with a0.0–0.5 and MSE with sunglasses.

To solve the issue of occlusion, especially the sunglasses, the normal face image has been augmented with an occluded image. For this purpose, an overlay over the eyes area with different alpha values (0.0–0.5) has been applied to augment the face image. Since the transparency of the overlay patch will affect the accuracy value it is important to select the optimal value of alpha for the experiments. By evaluating different alpha values, it is observed that the normal image and occluded image having alpha values 0.0 and 0.1 show high average accuracy values i.e. 96.5% for both as compared to other alpha values. It is also observed that the combination of different alpha values i.e. from 0.0 to 0.2 and 0.0 to 0.4 shows higher accuracy of 97%. The reason for not selecting this combination is to avoid more computational costs that are required for augmentation and training. To select the optimal alpha value we have calculated the mean square error of alpha values (0.0–0.5) as shown in Fig 6. The Mean Square Errors (MSE) of alpha values greater than zero are computed with the baseline of alpha value zero. If the error is small, then the image is closer to the baseline image otherwise it is different from the baseline. Hence, alpha values provided us with the baseline value for further experiments. All the presented experimental results are based on the alpha value of 0.0.

To demonstrate the experimental results with sunglasses, we have set three different conditions (Normal + both eyes, Normal+ Scarf, and Normal + both eyes + Scarf) as shown in Table 4. In this table, the Normal reflects the gallery image of Set A. Firstly, the probe images from H-J and U-W (termed sunglasses probe images) are tested against gallery images without any augmentation. In the next step, the sunglasses probe images are tested against augmented images of both eyes and gallery images. Later, sunglasses probe images are tested against augmented images of the scarf. At last, the sunglasses probe images were tested against the combination of eyes and scarf occluded images. From the results of the Session I (Normal + both eyes), it is noticed that high accuracy is achieved by set H i.e. 100%, whereas the lowest accuracy is obtained by set J i.e.96%. On the other hand, for Session II, U obtained higher accuracy i.e. 99% as compared to the W which obtained the lowest accuracy i.e. 91%. Similarly, when the Session I and II probe images are tested for normal+both eyes+scarf occlusion, it is observed that in Session I set H received higher accuracy i.e. 100%, and set w obtained lower accuracy i.e. 94%. However, for Session II, the higher accuracy is achieved by set U i.e. 98% and set W received lower accuracy i.e. 88%. The reason for declined accuracies for Normal+both eyes + scarf is the presence of facial images having scarf occlusion.

**Table 4:** Experimental results of occluded sunglasses set based on the accuracy.

Gallery Images	Probe Images					
	**Session I**			**Session II**		
	H	I	J	U	V	W
Normal	98%	93%	88%	97%	84%	85%
Normal +both eyes	100%	98%	96%	99%	95%	91%
Normal +both eyes + scarf	100%	96%	94%	98%	93%	88%

### 4.3 Occlusion with scarf

**Fig 7:**
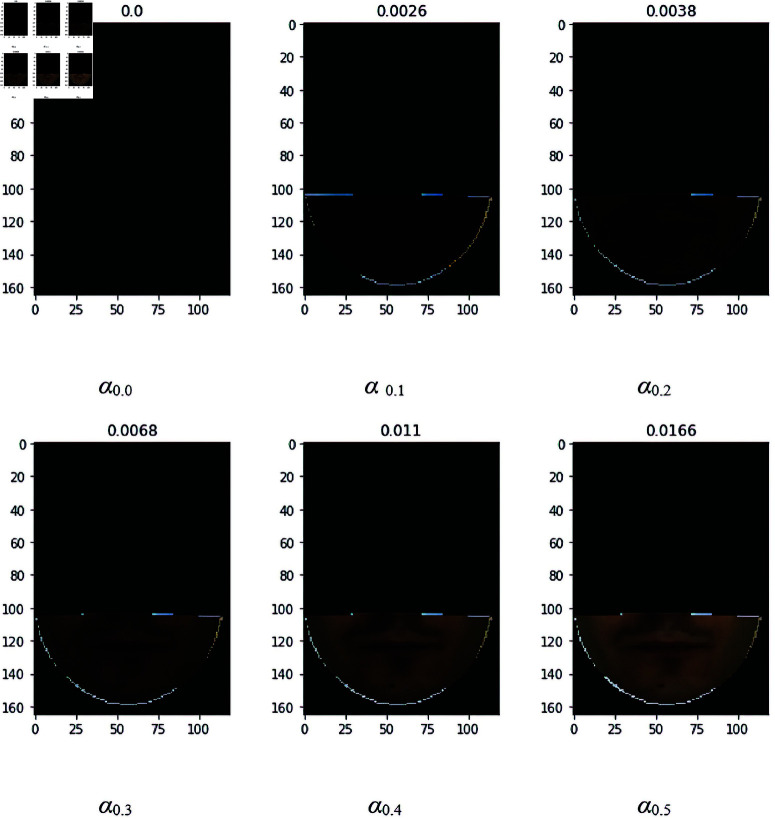
Occluded image with α 0.0–0.5 and MSE with scarf.

In order to solve the occlusion problem in the case of the scarf, the normal image has been tested with the occluded image. For this purpose, different α values (0.0–0.5) have been applied to the occluded image. For the overlay patch at the mouth, the transparency of the images is also evaluated based on the α value. It is observed that the normal image with the occluded image having α values 0.0 and 0.1 and 0.2 shows a high average accuracy value with 2 samples i.e. 99.17%, 99.17%, and 99.33% respectively. It is also observed that the combination of different α values i.e. from 0.0 to 0.3, 0.0 to 0.4, and 0.0 to 0.5 shows a higher accuracy of 99.50%. The reason for not selecting this combination is to avoid more computational costs that are required for augmentation and training. The analysis of Mean Square Error shows the α value 0.0 more transparency as shown in Fig 7. Hence for the experiments of occlusion with the scarf, the alpha value of 0.0 is considered a baseline value for further experiments.

While working occlusion with a scarf the sets K-M of Session I and X-Z of Session II are used for experimental results as shown in Table 5. The gallery image reflects the normal image. The probe images from K-M from Session I and X-Z from Session II (termed scarf probe images) are tested against gallery images without any augmentation. Then the scarf probe images are tested with augmented images of both eyes and gallery images. In the next step, the scarf probe images are tested against augmented images of the scarf and normal images. Lastly, the scarf probe images are tested against the combination of eyes and scarf occluded images along with normal images.

**Table 5:** Experimental results of occluded scarf set based on the accuracy.

Gallery Images	Probe Images					
	**Session I**			**Session II**		
	K	L	M	X	Y	Z
Normal	100%	100%	94%	99%	97%	95%
Normal+ Scarf	100%	100%	98%	100%	100%	98%
Normal +both eyes + scarf	100%	99%	96%	100%	97%	96%

During analysis of the results, it is noticed that the same accuracy was obtained by sets K and L of Session I for the Normal + Scarf occlusion i.e. 100% while Set M has 98% accuracy. Along the same lines, in Session II, sets X and Y achieved higher accuracy values i.e. 100%, and set Z received a 98% accuracy value. However, in normal+both eyes+scarf, the obtained results show that for Session I set K received a higher accuracy value i.e. 100%, and set M received a lower accuracy value i.e. 96%. For Session II, set X received 100% accuracy whereas set Z received lower accuracy i.e. 96%. The reason for the decline in accuracy for (normal+both eyes+scarf) is the presence of occluded images of sunglasses.

### 4.4 Misclassification results

Table 6 illustrates the misclassification with the subsets having facial images with sunglasses and scarf occlusion. For the sunglasses, the results indicate that in most of the subsets, the classification rate is less than 100% and higher than 90%. However, the V and W set to show the highest misclassification of 5% and 9% respectively. The reason for more classification is due to more exposure to light illumination and occlusion in terms of sunglasses. For the scarf occlusion, the results indicate that in most of the subsets, the classification rate is 100%. However, the M and Z sets show misclassification of 2% and 3% respectively. The misclassification results for local region-based augmentation techniques with scarf, sunglasses, and scarf+ sunglasses.

**Table 6:** Representation of results for comparison.

Misclassification	Datasets	Correct Prediction		Wrong Prediction	
		No. of Images	%	No. of Images	%
With sunglasses	H Set	100	100	0	0
	I Set	98	98.0	2	2.0
	J Set	96	96.0	4	4.0
	U Set	99	99.0	1	1.0
	V Set	95	95.0	5	5.0
	W Set	91	91.0	9	9.0
With scarf	K Set	100	100	0	0
	L Set	100	100	0	0
	M Set	98	98.0	2	2.0
	X Set	100	100	0	0
	Y Set	100	100	0	0
	Z Set	97	97.0	3	3.0

Table 7 illustrates the misclassification with the subsets having facial images with sunglasses + scarf occlusion. Here the results indicate that the highest classification rate is 100% for H, K, and X sets whereas the highest misclassification rate is for W sets i.e. 12%. It is observed here again the W set of Session II remains the most complex set.

**Table 7:** Misclassification with Scarf + Sunglasses.

Datasets	Correct Prediction		Wrong Prediction	
	No. of Images	%	No. of Images	%
H Set	100	100	0	0
I Set	96	96.0	4	4.0
J Set	94	94.0	6	6.0
K Set	100	100	0	0
L Set	99	99.0	1	1.0
M Set	96	96.0	4	4.0
U Set	98	98.0	2	2.0
V Set	93	93.0	7	7.0
W Set	88	88.0	12	12.0
X Set	100	100	0	0
Y Set	97	97.0	3	3.0
Z Set	96	96.0	4	4.0

## 5 Discussion

Local region-based augmentation provides the solution to improve the accuracy of SSPP FR by extending samples to add occlusions at the different regions of the face. For the comparison, we have set two protocols. In the first protocol, two studies are selected that have the same experimental setup [7] and [14] i.e. 100 subjects having 50 males and 50 females with 12 sets that deal with occlusion. In the second protocol, the obtained results are compared with the occlusion and light + occlusion with different existing studies. The results are compared with occlusion with sunglasses and scarf and light with occlusion of sunglasses and eyes. The comparison with existing studies shows the significance of the proposed local region-based augmentation technique as shown in Tables 8 and 9. The accuracy obtained by the proposed study is 94.83% for the occlusion with sunglasses, whereas it is 98% for scarf occlusion. The results obtained by the existing study [14] show better accuracy for sets I and J as compared to the present study for the occlusion with sunglasses, however for the occlusion with the scarf, and overall accuracy of the proposed approach shows better accuracy as compared to the existing study [14]. On the other hand, the proposed approach shows a significant improvement in accuracy compared to the existing research [7] i.e. 69.83% accuracy.

**Table 8:** Comparative analysis of existing techniques with sunglasses occlusion.

Research Studies	H	I	J	U	V	W	Average
DNNC[7]	97%	82%	78%	66%	54%	42%	69.83%
MB-C-BSIF[14]	100%	99%	98%	93%	81%	80%	91.83%
Proposed Work	100%	96%	94%	98%	93%	88%	94.83%

**Table 9:** Comparative analysis of existing techniques with scarf occlusion.

Research Studies	K	L	M	X	Y	Z	Average
DNNC [7]	88%	77%	70%	52%	47%	30%	60.67%
MB-C-BSIF[14]	99%	98%	96%	93%	87%	78%	91.67%
Proposed Work	100%	99%	96%	100%	97%	96%	98%

As mentioned earlier for protocol II, for a fair comparison with existing studies, we have merged H+K which is occlusion with sunglasses and eyes, and J+M which is light with occlusion of scarf and eyes. All these sets are taken from Session I. The comparison with existing studies shows the significance of the proposed local region-based augmentation technique as shown in Table 10. The obtained average accuracy for the sets H, and K is 100% in our case whereas for the sets J and M it was 97%. The comparison of results shows that the proposed study has declined slightly in accuracy as compared to the existing study [14] and [13]. The reason for better accuracy in [14] and [13] for light + occlusion is due to improved accuracy in the M set of Session I as compared to the proposed approach.

**Table 10:** Comparison with Session I (occlusion and light + occlusion)

Research Studies	No of Subjects	Occlusion H+K	Light + Occlusion J+M	Average Accuracy
MFSA [15]	100	91.5%	-	91.5%
SSLD [6]	100	90.18%	82.02%	86.1%
MB-C-BSIF [14]	100	99.50%	98.5%	99%
DNNC [7]	100	92.50%	79.5%	86%
BDL [19]	-	93.03%	91.55%	92.29%
MB-C-BSIF [13]	100 (50 male and 50 female)	99.66%	98.83%	99.25%
Proposed	100	100%	97%	98.5%
Approach				

Occlusion over the upper face like covering the eyes and nose affects the accuracy of the face recognition system as compared to the occlusion of the lower face like a scarf or mask that covers the mouth or chin. This is evident from the experiments without introducing the occlusion augmentation to the gallery image. The results shown in Table 11 reveal that for the sunglasses occlusion, the gallery images having normal + both eyes occlusion obtained a 5.7% improvement. However, the gallery images having normal+ scarf occlusion obtained declined accuracy by -15.8%. Due to the fact of a different region of occluded parts on the face. For the scarf occlusion, it was noticed that the gallery images with the scarf and with both eyes and scarf had obtained an improvement of 1.67% and 0.5% respectively. The decline observed was -8% for the gallery images with both eyes.

**Table 11:** Representation of results for comparison.

Gallery Images	Sunglasses (90.8)		Scarf (97.5)	
	Average Value	Improvement	Average Value	Improvement
Normal + both eyes	96.5%	5.7↑	89.5%	-8↓
Normal +Scarf	75%	-15.8↓	99.33%	1.8↑
Normal +both eyes + scarf	94.83%	4.03↑	98%	0.5↑

### 5.1 Limitations of the study

Unlike normal face recognition, SSPP face recognition relies on a single image that is taken in a controlled environment with proper light and resolution. It does not include the other variations available in an unconstrained environment. Hence some limitations are highlighted while working with the dataset available for SSPP face recognition.

The major limitation of working with SSPP face recognition datasets is that most of the datasets are not specifically designed for SSPP FR. The training sample also has some challenges to deal with. Another limitation is that computer vision and CNN techniques require high-quality testing images so it will be hard to work with low-quality, very dark, and occluded images. Due to the insufficient available data for training, the performance of the proposed approach was evaluated using the pre-trained model. We also analyzed that there is no publicly available dataset to represent exactly the Single Sample Per Person problem that could apply to real-time scenarios such as Law enforcement and e-passport problems.

Further, in the proposed study, large datasets or real-time scenario-based datasets were not used. Due to the limitations of existing techniques for varied capturing conditions, for the evaluation of the efficiency of the proposed approaches for occlusion, there is a requirement for a real-time dataset that should be captured in different scenarios.

## 6 Conclusion

To address the occlusion variance, a local region-based augmentation technique has been applied. Landmark key points are used to extract the key points of the face. Based on these key points, the region of interest is extracted from a particular region. An overlay patch is applied to the region of interest to create facial images with artificial occlusion. These occluded faces are used to create virtual samples. We have used these virtual samples to train the model. Although we focused on occlusions caused by sunglasses and scarves, our methodology can be directly extended to other sources of occlusion such as hats, beards, and long hair. We have shown that the extraction of landmark points from the local area can increase the accuracy of classifiers. The proposed approach requires a small number of augmented training samples and hence has the advantage of minimizing the training overhead of the classifier models. Despite the promising results, our approach has certain limitations. The study primarily focuses on occlusions caused by sunglasses and scarves, while other types of occlusions, such as hand occlusions, and dynamic occlusions in videos, were not extensively considered. Additionally, the evaluation was conducted on limited datasets, and the generalizability of the proposed method to large-scale and more diverse datasets remains unverified. Furthermore, the approach is designed for static images, and its effectiveness in handling occlusions in real-time video-based applications has not been explored. Practical deployment in real-world scenarios may also introduce computational challenges, such as processing speed and adaptability to varying environmental conditions. Future research on single sample per person face recognition, particularly in handling occlusions and recognizing individuals, can benefit from several advanced techniques. Self-supervised learning and transformer-based models, such as Vision Transformers and Masked Autoencoders, can improve feature extraction and help the system recognize faces even when parts are obscured. Diffusion models can be used to restore missing facial details and generate additional training samples, improving robustness. Graph Neural Networks can analyze facial landmarks to focus on visible regions, making recognition more reliable.
